# CD4+Foxp3+ regulatory T cells prolong drug-induced disease remission in (NZBxNZW) F1 lupus mice

**DOI:** 10.1186/ar4188

**Published:** 2013-02-27

**Authors:** Olivia Weigert, Caroline von Spee, Reinmar Undeutsch, Lutz Kloke, Jens Y Humrich, Gabriela Riemekasten

**Affiliations:** 1 Department of Rheumatology and Clinical Immunology, Charité - University Hospital, Charitéplatz 1, 10117 Berlin, Germany

## Abstract

**Introduction:**

The ability to ameliorate murine lupus renders regulatory T cells (Treg) a promising tool for the treatment of systemic lupus erythematosus (SLE). In consideration to the clinical translation of a Treg-based immunotherapy of SLE, we explored the potential of CD4+Foxp3+ Treg to maintain disease remission after induction of remission with an established cyclophosphamide (CTX) regimen in lupus-prone (NZBxNZW) F1 mice. As a prerequisite for this combined therapy, we also investigated the impact of CTX on the biology of endogenous Treg and conventional CD4+ T cells (Tcon).

**Methods:**

Remission of disease was induced in diseased (NZBxNZW) F1 mice with an established CTX regimen consisting of a single dose of glucocorticosteroids followed by five day course with daily injections of CTX. Five days after the last CTX injection, differing amounts of purified CD4+Foxp3+CD25+ Treg were adoptively transferred and clinical parameters, autoantibody titers, the survival and changes in peripheral blood lymphocyte subsets were determined at different time points during the study. The influence of CTX on the numbers, frequencies and proliferation of endogenous Treg and Tcon was analyzed in lymphoid organs by flow cytometry.

**Results:**

Apart from abrogating the proliferation of Tcon, we found that treatment with CTX induced also a significant inhibition of Treg proliferation and a decline in Treg numbers in lymphoid organs. Additional adoptive transfer of 1.5 × 10^6 ^purified Treg after the CTX regimen significantly increased the survival and prolonged the interval of remission by approximately five weeks compared to mice that received only the CTX regimen. The additional clinical amelioration was associated with an increase in the Treg frequency in the peripheral blood indicating a compensation of CTX-induced Treg deficiency by the Treg transfer.

**Conclusions:**

Treg were capable to prolong the interval of remission induced by conventional cytostatic drugs. This study provides valuable information and a first proof-of-concept for the feasibility of a Treg-based immunotherapy in the maintenance of disease remission in SLE.

## Introduction

Systemic lupus erythematosus (SLE) is a prototypic systemic autoimmune disease characterized by the breach of tolerance to ubiquitous nuclear self-antigens, resulting in an uncontrolled activation of the immune system at both a cellular and a humoral level that leads to multi-organ inflammation, most importantly nephritis [1-5].

Evidence for the effective treatment of SLE exists for glucocorticosteroids (GC) and immunosuppressive drugs [2,6,7]. In addition, monthly pulses of the cytostatic agent cyclophosphamide (CTX) are commonly applied in combination with high doses of GC to induce remission of severe disease and active nephritis [8-10]. Immunosuppressive treatments are usually very effective but can be accompanied by severe adverse events, infections and toxicity, especially when applied over a longer period of time [11]. This has become a major prognostic issue nowadays. In view of that, the search for more specific therapeutic strategies to minimize side effects and to allow a better quality of life for the patients is an important focus of current research efforts.

Regulatory T cells (Treg), expressing the transcription factor Foxp3, are crucial for the maintenance of peripheral self-tolerance [12-14]. Their unique capacity to prevent autoimmunity renders CD4+Foxp3+ Treg an attractive tool for the treatment and modulation of autoimmune diseases. Cellular immunotherapy of autoimmune diseases intends to restore the disturbed tolerance to self by transfer of *ex vivo *expanded autologous Treg that can inhibit the activation and expansion of effector T cells [15-20].

In human and murine lupus, a deficiency, and phenotypic abnormalities of Treg are evident, suggesting that a disturbance of the Treg system is involved in disease development [21-28]. In the (NZBxNZW) F1 mouse model for lupus we previously found that a progressive and self-amplifying disruption of Treg homeostasis due to an acquired IL-2 deficiency essentially contributes to the hyperactivity of conventional CD4+ T cells (Tcon) and disease development [21]. In addition, we demonstrated the general importance of CD4+Foxp3+ Treg as physiologically relevant inhibitors of lupus by several approaches. First, the reduction of Treg numbers in clinically healthy animals by antibody-mediated depletion of CD25+ cells, or by IL-2 neutralization, resulted in an acceleration of disease. Second, and in contrast, adoptive transfer of CD4+Foxp3+CD25+ Treg-delayed disease progression and significantly reduced mortality in mice with already established disease [21], indicating that, depending on a sufficient amount, Treg are able to counteract even chronically active autoimmunity. In addition, we found that Treg from both young, clinically healthy, and diseased (NZBxNZW) F1 mice had a similar suppressive capacity in comparison to Treg from age-matched BALB/c mice [21], ruling out an intrinsic functional Treg defect in murine lupus. Thus, therapeutic strategies that pursue the reconstitution of Treg-mediated peripheral tolerance by increasing the pool size of Treg are very promising for the treatment of SLE, and the clinical applicability of such an approach is well worth investigating in more detail.

With regard to a clinical application, the sequential combination of Treg-based immunotherapy with conventional therapeutic and immunosuppressive approaches could be advantageous. Mono-therapy with Treg alone may not be sufficient to induce a vigorous and long-lasting remission in chronic autoimmune diseases because of the strong cellular and humoral activity and the pre-existence of a robust immunological memory against self-antigens, especially in SLE [21,28-32]. Inhibition of the activation and expansion of pathogenic cells by immunosuppressive or cytostatic drugs prior to the intended Treg transfer may therefore increase their therapeutic efficacy by creating synergistic conditions where Treg are better capable of keeping autoreactive conventional T cells (Tcon) and other pathogenic cells under control. Accordingly, the therapeutic relevance of Treg could be in maintaining remission after induction of remission with conventional immunosuppressive drugs, thereby enabling a dose reduction, or even discontinuation of these drugs, and thus, avoiding long-term drug-related side effects and toxicities.

The aim of our current study was to adapt the previous Treg-based mono-therapy from experimental to more applicable clinical settings by combining a conventional GC/CTX regimen, known to be very effective also in lupus-prone animals [33-35], with a subsequent adoptive transfer of Treg in the (NZBxNZW) F1 mouse model for lupus. As a prerequisite for this combined therapy, we also investigated the impact of the cytostatic agent CTX on the biology of pre-existing, endogenous Treg.

## Materials and methods

### Mice

Female (NZBxNZW) F1 mice were obtained from the breeding facility of the Deutsches Rheuma-Forschungszentrum (DRFZ, Berlin, Germany). All mice were bred and maintained under specific pathogen-free conditions in the DRFZ and were used for experiments between 6 weeks and 7 months of age. All experiments were performed according to institutional and federal guidelines (Landesamt für Gesundheit und Soziales, LAGeSo, Berlin, Germany).

### Flow cytometry

Cells were stained with the indicated antibodies in PBS containing 0.2% BSA and 0.01% sodium azide. The following conjugated antibodies to mouse antigens were generated in the DRFZ: anti-CD4-biotin (clone GK 1.5), anti-CD4-phycoerythrin (PE) (clone GK 1.5), anti-CD62L-biotin (clone MEL-14), anti-CD44-fluorescein isothiocyanate (FITC) (clone IM7), and anti-CD3-PE (clone 145-2C11). The following antibodies and secondary reagents were purchased from the indicated manufacturers: anti-CD25-allophycocyanin (APC) (BD Biosciences, Heidelberg, Germany, clone PC61), anti-CD19-APC (BD Biosciences, clone 1D3), anti-IgD-FITC (BD Biosciences, clone 11-26c.2a), anti-CD138-PE (BD Biosciences, Syndecan-1, clone 281-2), anti-CD19-APC (BD Biosciences, clone 1D3), Streptavidin- Peridinin-Chlorophyll-Protein Complex (PerCP) (BD Biosciences), anti-CD69-FITC (BD Biosciences, clone H1.2F3), anti-CD25-PE (Miltenyi Biotec GmbH, Bergisch Gladbach, Germany, clone 7D4), anti-CD4-FITC and anti-CD4 PerCP (BD Biosciences, clone RM4-5/L3T4). For the intracellular detection of Foxp3, FITC-, PE- and APC-conjugated anti-Foxp3 antibodies (eBioscience, Frankfurt, Germany, clone FJK-16s) were used with the appropriate buffers according to the manufacturer´s protocol. Absolute cell numbers in a preparation were determined from the ratio of cells to microbeads (Fluoresbrite^® ^YG microspheres 20µm, Polysciences Europe GmbH, Eppelheim, Germany) in a defined volume; these numbers were used to calculate the total number of cells in lymphoid organs and in the peripheral blood. Stained cells were analyzed on a FACSCalibur cytometer (BD Biosciences). Results were processed using FlowJo software (Tree Star, Inc., OR, USA). FACSDiva (BD Biosciences) and FACSAria (BD Biosciences) cell sorters were used to purify cell populations.

### Assessment of *in vivo *proliferation by 5-Bromo-2'-deoxyuridine (BrdU) labeling

(NZBxNZW) F1 mice with established disease (proteinuria ≥100 mg/dl) were treated intravenously every 24 h with CTX (30 µg/g bodyweight) for a total of seven times, and in parallel, were injected intraperitoneally every 24 h with BrdU (40 µg/g bodyweight) (BD Biosciences) for a total of four times during the last 4 days of the CTX regimen. Mice were sacrificed 12 h after the last injection and cells from lymphoid organs and the peripheral blood were separately isolated for further analysis by flow cytometry. BrdU incorporation was detected by intracellular staining with anti-BrdU-FITC antibodies according to the manufacturer´s protocol (BrdU Flow kit, BD Biosciences). Intracellular staining for Foxp3 was performed in parallel to the BrdU staining with PE-conjugated anti-Foxp3 antibodies (eBioscience). Surface staining with anti-CD4 antibodies was performed before the fixation of cells.

### Induction of disease remission with GC/CTX

(NZBxNZW) F1 mice with established disease at the age between 6 and 7 months and proteinuria ≥100 mg/dl were initially treated intravenously with a single dose of 10 µg/g bodyweight of prednisolone (Solu-Decortin H; Merck Pharma, Darmstadt, Germany). Then CTX (Pharmacy of Charité, Berlin, Germany) was given intravenously at a dose of either 20 or 30 µg/g bodyweight every 24 h for a total of five times.

### Adoptive transfer of Treg

**Figure 1:**
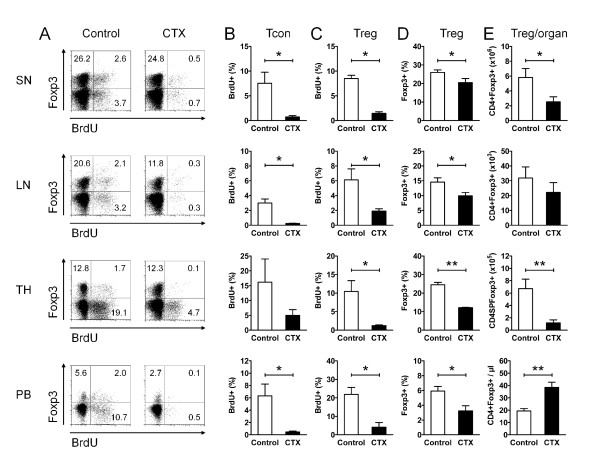
**Cyclophosphamide (CTX) inhibits regulatory T cell (Treg) proliferation and causes a gap in Treg numbers**. (**A**-**E**) Flow cytometry of CD4+Foxp3+ Treg and CD4+Foxp3- conventional T cells (Tcon) in the spleen (SN), lymph nodes (LN), thymus (TH) and peripheral blood (PB) of CTX-treated (CTX) (NZBxNZW) F1 mice with active disease compared to age-matched PBS-treated controls (Control). (**A**) Representative dot plots show 5-Bromo-2'-deoxyuridine (BrdU) incorporation of CD4+Foxp3+ Treg and of CD4+Foxp3- Tcon from CTX-treated and control mice in the respective compartments. The numbers in the quadrants indicate the percentage of the respective population among CD4+ cells. (**B**, **C**) Average percentage of BrdU+ cells among CD4+Foxp3- Tcon (**B**) and among CD4+Foxp3+ Treg (**C**). Data represent the means of four to five mice per group from two independent experiments. (**D**, **E**) Average percentage of CD4+Foxp3+ Treg among CD4+ T cells (**D**) and average absolute counts of CD4+Foxp3+ Treg in the respective organ (**E**). Data represent the means of five to ten mice per group from several independent experiments. (**B**-**E**) Error bars indicate standard error of the mean (SEM) (*P <0.05, **P <0.01, CTX vs Control).

Donor cells were obtained from lymph nodes and spleens of (NZBxNZW) F1 mice between 6 to 10 weeks of age. CD4+ T cells were enriched with a CD4+ T cell isolation kit by negative selection (Miltenyi Biotec). Unlabelled CD4+ T cells were further stained with anti-CD4-FITC (BD Biosciences, clone RM4-5,) and anti-CD25-PE (Miltenyi Biotec, clone 7D4) and CD4+CD25+ Treg were purified by cell sorting. The purity of isolated CD4+CD25+ cells was greater than 95% and more than 95% of sorted CD4+CD25+ cells expressed Foxp3 (see also Figure 1B). Sorted Treg were incubated at 37° in cell culture medium (Roswell Park Memorial Institute (RPMI) 1640 with 10% FCS and penicillin/streptomycin) supplemented with 40 ng/ml of recombinant mouse IL-2 (rmIL-2, R&D Systems GmbH, Wiesbaden, Germany) for 4 h. Then cells were harvested, washed twice with PBS, and either 0.5 or 1.5 × 10^6 ^cells suspended in PBS were injected intravenously into (NZBxNZW) F1 mice five days after the last injection of CTX. Controls received an equal amount of PBS after treatment with GC/CTX.

### Monitoring of disease activity

Proteinuria and leukocyturia were determined with Multistix 10 Visual (Siemens Healthcare Diagnostics Inc., Tarrytown, NY, USA). The following scoring system was used: score 0 = 0 to 15 mg/dl, score 1 = 30 mg/dl, score 2 = 100 mg/dl, score 4 = 300 mg/dl, score 6 = 2,000 mg/dl. A score of 6 was maintained for mice that died during the experiment.

### Monitoring of cells in the peripheral blood

For the analysis of peripheral blood lymphocytes, 50 to 100 µl of whole blood was sampled from the tail vein of each animal before (day -10) and after the GC/CTX treatment (day -4), and every two weeks during follow up after the Treg transfer (at day 0). Blood clotting was prevented using heparin (Ratiopharm GmbH, Ulm, Germany)-containing vials. Plasma was separated from the blood samples and stored at -20°C until further analysis. Erythrocytes were lysed using a standard lysis buffer. All cell suspensions were dissolved in PBS containing 0.2% BSA and were immediately used for flow cytometry.

### ELISA

The concentration of anti-ds-DNA antibodies in the plasma was determined by ELISA according to standard procedures [36].

### Statistical analysis

Graph Pad Prism 5 software was used for the analysis of survival curves (log rank test, Kaplan-Meier curve). The Mann-Whitney test and when appropriate, the Wilcoxon signed rank test was used to detect statistically significant differences. *P*-values less than 0.05 were regarded significant.

## Results

### CTX inhibits Treg proliferation and causes a gap in Treg numbers

As a precondition for the sequential therapeutic approach with CTX and Treg, we explored the impact of the cytostatic agent CTX on the biology and homeostasis of endogenous CD4+Foxp3+ Treg in comparison to CD4+Foxp3- Tcon in our disease model. Absolute numbers, frequencies and *in vivo *proliferation rates of CD4+Foxp3+ Treg and CD4+Foxp3- Tcon were determined in lymphoid organs and the peripheral blood by BrdU incorporation during a seven-day treatment course with CTX in (NZBxNZW) F1 mice with established disease.

Treatment with CTX induced almost complete inhibition of Tcon proliferation in the spleen, lymph nodes, thymus and peripheral blood (Figure 1A,B) resulting in a decrease in the absolute numbers of Tcon with the exception of the peripheral blood, where CTX induced an increase in the frequency and the absolute numbers of all CD4+ T cells (mean percentage of CD4+ T cells among lymphocytes 29.3 % vs 9.2%, *P *= 0.001; mean absolute number of CD4+ T cells 1043/µl vs 418/µl, *P *= 0.0013, for CTX vs control, respectively) (data not shown). In parallel, the proliferation of Treg was almost similarly inhibited by CTX (Figure 1A,C). This was accompanied by a reduction in the absolute numbers and also in the frequencies of CD4+Foxp3+ Treg in the lymphoid organs (Figure 1D,E). In the peripheral blood, CTX also induced a decrease in the percentage of Foxp3+ Treg among CD4+ T cells (Figure 1D); however, there was a significant increase in absolute numbers of CD4+Foxp3+ Treg (Figure 1E) due to the distinctive increase in the frequency and absolute numbers of all CD4+ T cells among peripheral blood lymphocytes. Together, CTX induced a diminution of the Treg pool size in lymphoid organs most likely by inhibiting the homeostatic proliferation of Treg. This important interference of CTX with endogenous Treg was taken into account with regard to the design of the combined, sequential therapeutic approach consisting of a CTX regimen and Treg transfer.

### Adoptive Treg transfer prolongs drug-induced disease remission

**Figure 2:**
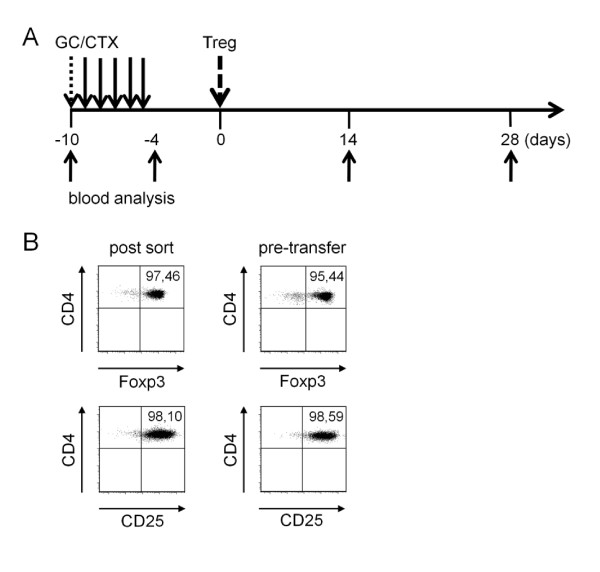
**Treatment schedule and regulatory T cell (Treg) purification**. **(A**) (NZBxNZW) F1 mice with active disease received 10 µg/g body weight of prednisolone (GC) intravenously at day -10 followed by daily intravenous injections of either 20 or 30 µg/g body weight of CTX for the duration of 5 days. Another 5 days later, CD4+Foxp3+CD25+ Treg were purified from the lymphoid organs of young (NZBxNZW) F1 mice, cultured for 4 h in the presence of 40 ng/ml of rIL-2 and either 0.5 × 10^6 ^or 1.5 × 10^6 ^Treg were adoptively transferred by intravenous injection at day 0. Control mice were equally treated with GC/CTX and received PBS instead of Treg. Blood samples and plasma were collected at the indicated time points. (**B**) Representative dot plots show the expression of Foxp3 and CD25 of CD4+CD25+ sorted donor Treg briefly after the sorting procedure (left, post sort) and just before the adoptive cell transfer (right, pre-transfer).

Next, we investigated the capability of Treg to prolong disease remission after induction of remission in lupus-prone mice. To induce remission of active disease prior to the intended transfer of Treg, diseased (NZBxNZW) F1 mice were treated once with GC followed by daily intravenous injections of CTX for a total of five days, as outlined in Figure 2A. Treg were isolated from spleens and lymph nodes of young (NZBxNZW) F1 mice by sorting for CD4+CD25+ cells. The purity of sorted Treg reached up to 95% determined by the expression of Foxp3 among sorted CD4+CD25+ T cells, indicating that the large majority of sorted cells belonged to the Treg lineage (Figure 2B). Purified Treg were cultured for 4 h in the presence of rIL-2 before the adoptive transfer by intravenous injection. During this short-term culture, expression levels of Foxp3 and CD25 were maintained indicating a stable Treg phenotype immediately before transfer (Figure 2B). To avoid interference between CTX and the transferred Treg in the recipients, Treg were transferred five days after the last CTX injection (day 0) ensuring that most of the CTX was already cleared from the body of the recipients according to its pharmacological half-life of approximately 6 to 7 h [37,38].

**Figure 3:**
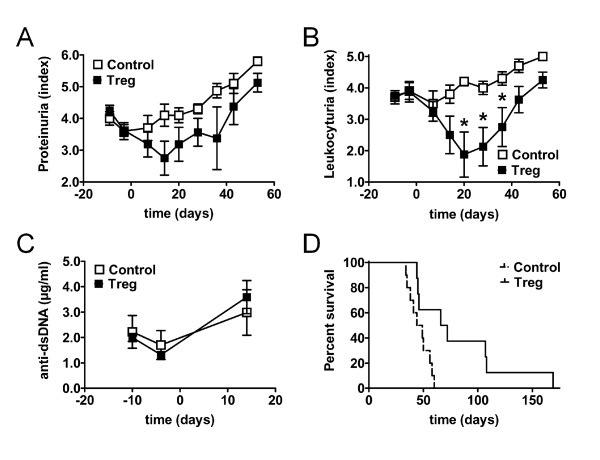
**Regulatory T cells (Treg) prolong drug-induced disease remission**. **(A**-**D**) Changes in clinical parameters and survival of (NZBxNZW) F1 mice with active disease after induction of remission with glucocorticosteroid (GC)/cyclophosphamide (CTX) and after an additional adoptive transfer of 1.5 × 10^6 ^Treg/mouse (Treg) compared to age-matched mice that received only GC/CTX and PBS (Control). (**A**-**C**) Average proteinuria score (**A**), leukocyturia score (**B**) and levels of antibodies against ds-DNA (**C**) determined at the indicated time points during the study. Error bars indicate standard error of the mean (SEM) (*P <0.05; Treg vs Control at the indicated time point). (**D**) Survival time is presented as a Kaplan-Meier curve (*P *= 0.01; Treg vs Control). (**A**-**D**) Data are the summary of two to three independent experiments, with eight to ten mice per group in total.

Treatment with GC/CTX alone induced remission of disease shown by the transient decrease in proteinuria (day -10 vs day -4, *P *<0.001) (Figure 3A). However, leukocyturia was not affected by the GC/CTX treatment (Figure 3B). In addition, a transient decrease in the levels of antibodies against dsDNA was observed (day -10 vs day - 4, *P *<0.001) (Figure 3C). Proteinuria and anti-dsDNA antibodies returned to pre-treatment levels approximately three weeks after the last CTX injection (Figure 3A,C, control).

Adoptive transfer of 0.5 × 10^6 ^Treg per mouse five days after the last CTX injection did not additionally affect proteinuria or leukocyturia (data not shown), and resulted in no considerable change in the mortality rate compared to control group (*P *= 0.38) (data not shown). Thus, we performed similar experiments with a 3-fold higher amount of transferred Treg. Adoptive transfer of 1.5 × 10^6 ^Treg per mouse five days after the GC/CTX regimen resulted in a moderate additional reduction of proteinuria (Treg vs control, *P *= 0.06 at day 14 after transfer) (Figure 3A). In contrast to the treatment with GC/CTX, additional Treg transfer now considerably affected leukocyturia shown by the significant reduction of leukocyturia from day 20 to day 36 after transfer compared to the treatment with GC/CTX alone (Treg vs control, *P *<0.05) (Figure 3B). However, no additional reduction in the levels of auto-antibodies could be detected (Figure 3C), consistent with previous observations after Treg transfers in this model [21,26]. Most importantly, mice that received a high dose of Treg had a significantly decreased mortality rate compared to the control group (Treg vs control median survival 69 vs 46 days, *P *= 0.01) (Figure 3D). Thus, Treg were able to prolong disease remission induced by a conventional immunosuppressive regimen. The additive effect of Treg on the GC/CTX regimen, however, depended on a high number of transferred Treg.

### Prolongation of disease remission is associated with an increase in the Treg frequency

To obtain insights into possible effects of Treg therapy at a cellular level we determined the percentage, absolute numbers and the phenotype of T and B cell subsets in the peripheral blood by flow cytometry before (day -10) and after induction of remission (day -4), and every 14 days after the Treg transfer (at day 0).

**Figure 4:**
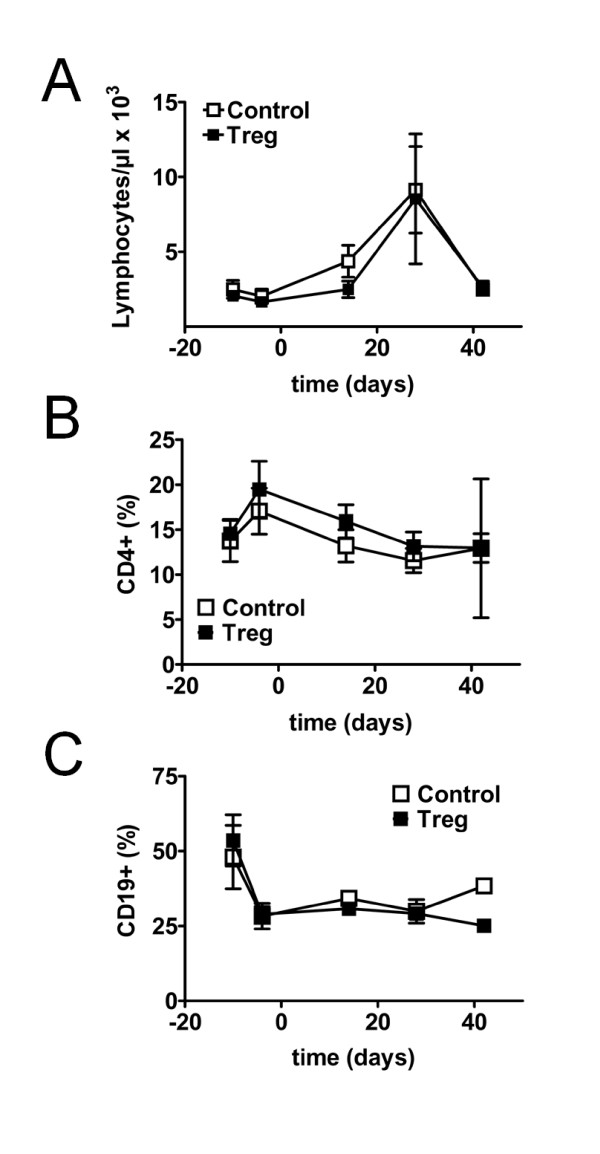
**Changes in lymphocyte subsets during induction of remission and sequential regulatory T cell (Treg) transfer**. Average absolute numbers of total lymphocytes (**A**) and average percentage of CD4+ T cells (**B**) and CD19+ B cells (**C**) among lymphocytes in the peripheral blood of (NZBxNZW) F1 mice with active disease before and after induction of remission with glucocorticosteroid (GC)/cyclophosphamide (CTX), and after an additional adoptive transfer of 1.5 × 10^6 ^Treg/mouse (Treg) compared to age-matched mice that received only GC/CTX and PBS (Control). Data are the summary of two independent experiments with six to eight mice per group in total. Error bars indicate standard error of the mean (SEM).

Treatment with GC/CTX alone induced a late and transient increase in the numbers of total lymphocytes in the peripheral blood (Figure 4A). Consistent with prior data, the percentage of CD4+ T cells among total lymphocytes transiently increased after the CTX regimen (day -10 vs day -4, *P *<0.001) (Figure 4B). In contrast, there was a dramatic decrease in the percentage of CD19+ B cells among lymphocytes (*P *<0.001) that persisted throughout the observation time (Figure 4C). However, no differences in total lymphocytes, CD4+ T cells or CD19+ B cells after additional adoptive transfer of Treg could be observed compared to mice that received only GC/CTX (Figure 4 A-C).

**Figure 5:**
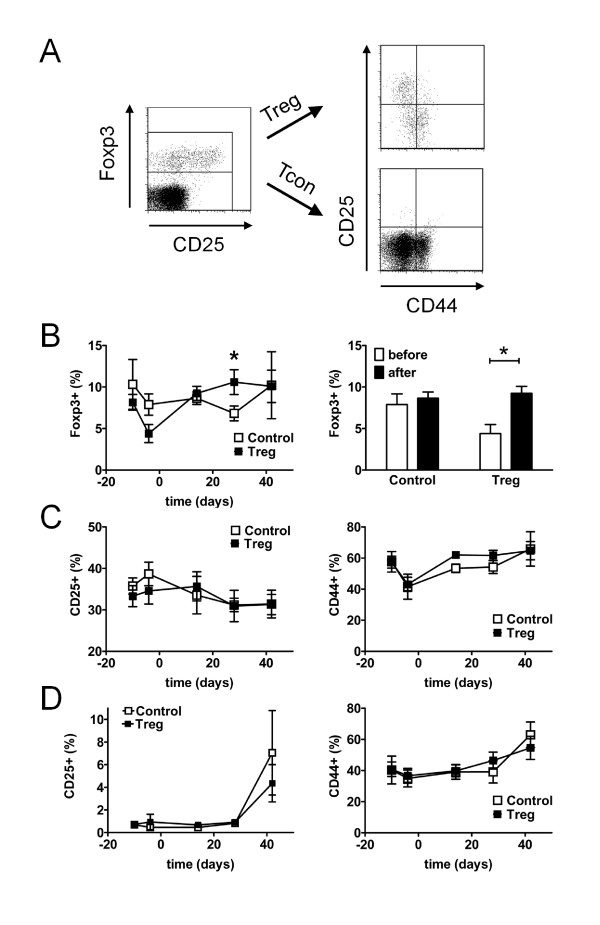
**Changes in regulatory T cell (Treg) and conventional T cell (Tcon) subsets during induction of remission and sequential Treg transfer**. Treg and Tcon subsets were analyzed by flow cytometry in the peripheral blood of (NZBxNZW) F1 mice during induction of remission and after an additional adoptive transfer of 1.5 × 10^6 ^Treg/mouse (Treg) and compared to age-matched mice that received only glucocorticosteroid (GC)/cyclophosphamide (CTX) and PBS (Control). (**A**) Representative dot plots show expression of CD25 and CD44 among CD4+ gated Foxp3+ Treg and Foxp3- Tcon. (**B**) Average percentage of Foxp3+ Treg among CD4+ T cells at the indicated time points during the course of the study (*P <0.05; Treg vs Control). Bar diagrams compare the average percentage of Foxp3+ Treg among CD4+ T cells before (at day -4) and after the Treg transfer (at day 14) within the respective group (*P <0.05; before vs after). (**C**) Average percentage of CD25+ cells and CD44hi cells among CD4+Foxp3+ Treg at the indicated time points during the study. (**D**) Average percentage of CD25+ cells and CD44hi cells among CD4+Foxp3- Tcon throughout the study. (**B**-**D**) Data are the summary of two independent experiments with six to eight mice per group in total. Error bars indicate standard error of the mean (SEM).

The percentage and phenotype of CD4+Foxp3+ Treg and of CD4+Foxp3- Tcon was analyzed as outlined in Figure 5A. Consistent with our previous observations, treatment with GC/CTX led to a significant decrease in the percentage of Foxp3+ Treg among CD4+ T cells in the peripheral blood (day -10 vs day -4, *P *<0.01) (Figure 5B). However, mice that received Treg had a significantly higher frequency of CD4+Foxp3+ Treg at day 28 compared to control mice (*P *<0.05) (Figure 5B). In addition, by separately comparing the frequencies of CD4+Foxp3+ Treg before (day -4) and after (day 14) transfer in each treatment group, we observed a 2-fold increase in Treg frequency in mice that received Treg (*P *<0.05), whereas in mice that were treated with GC/CTX alone Treg frequencies remained unaffected (Figure 5B). Thus, adoptive transfer of Treg led to an increase in Treg in the recipients and compensated the CTX-induced gap in Treg numbers.

The percentage of CD25+ cells among CD4+Foxp3+ Treg was neither affected by GC/CTX treatment nor by the additional Treg transfer (Figure 5C). The percentage of CD44hi memory cells among Foxp3+ Treg initially declined after the GC/CTX treatment (*P *<0.05) but quickly returned to pre-treatment levels independent of the additional Treg transfer (Figure 5C). Analysis of CD44hi memory/effector cells and of CD25+ cells among CD4+Foxp3- Tcon revealed no significant differences in the percentages between both treatment groups at any time point throughout the study (Figure 5D).

**Figure 6:**
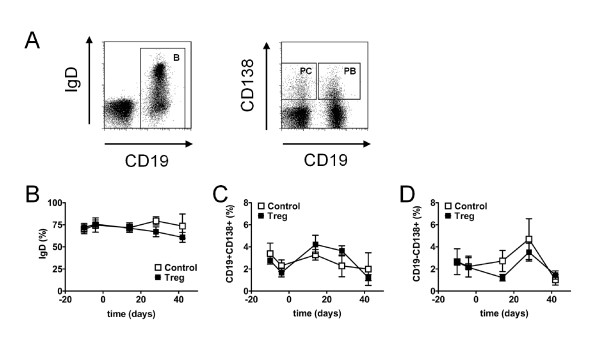
**Changes in B cell subsets during induction of remission and sequential regulatory T cell (Treg) transfer**. B cell subsets were analyzed by flow cytometry in the peripheral blood during induction of remission and after an additional adoptive transfer of 1.5 × 10^6 ^Treg/mouse (Treg) and compared to age-matched mice that received only glucocorticosteroid (GC)/cyclophosphamide (CTX) and PBS (Control). (**A**) Representative dot plots show expression of IgD among CD19+ B cells and the percentage of CD19+CD138+ plasma blasts (PB) and of CD19-CD138+ plasma cells (PC) among lymphocytes, respectively. (**B**-**D**) Average percentage of IgD+ cells among CD19+ B cells (**B**), average percentage of CD19+CD138+ PB among lymphocytes (**C**) and average percentage of CD19-CD138+ PC among lymphocytes (**D**) at the indicated time points during the study. Data are the summary of two independent experiments with six to eight mice per group in total. Error bars indicate standard error of the mean (SEM).

B cell subsets were analyzed and gated as displayed in Figure 6A. Neither the GC/CTX treatment nor the additional Treg transfer significantly affected the percentage of IgD+ naive B cells among CD19+ B cells (Figure 6B). The frequency of CD19+CD138+ plasma blasts (PB) among total lymphocytes transiently decreased after the GC/CTX treatment (*P *<0.05) but returned to pre-treatment levels approximately three weeks after the GC/CTX treatment with no obvious difference between both groups (Figure 6C). Interestingly, we observed that the additional Treg transfer further enhanced the slight reduction in the frequency of CD19-CD138+ plasma cells (PC) induced by GC/CTX that even reached a significant level when comparing baseline values at day -10 with the values obtained at day 14, after transfer within the Treg group (*P *<0.05) (Figure 6D).

## Discussion

The potential of CD4+Foxp3+ Treg to influence the course of immune-mediated disorders and autoimmune diseases has become a major focus of advanced cellular immunotherapy. In consideration of future clinical translation of Treg-based immunotherapy in SLE, we explored the potential of adoptively transferred Treg to maintain disease remission after induction of remission by a conventional immunosuppressive approach in the (NZBxNZW) F1 mouse model for lupus.

CTX is one of the most commonly used cytostatic agents to induce remission of severe disease in SLE patients and was shown to be effective also in lupus-prone animals [33-35]. Recently, several groups reported that a low-dose regimen of CTX can affect CD4+Foxp3+ Treg in humans by decreasing their pool size or by inhibiting their functionality [39-44]. Thus, we aimed to obtain insights into the cellular situation that is found after CTX treatment and prior to an adoptive transfer of Treg in the (NZBxNZW) F1 mouse model. For the induction of remission in this disease model a repetitive high dose regimen of CTX has been proven to be effective [33-35]. In our settings, we found that CTX induced a virtually complete inhibition of the proliferation of CD4+ Tcon in lymphoid organs and the peripheral blood of lupus mice. This is consistent with the notion that the beneficial effect of CTX is associated with an inhibition of the expansion of activated effector T cells that contribute to disease pathology [21,31,45]. However, the proliferation of CD4+Foxp3+ Treg was found to be almost equally inhibited resulting in a profound reduction in the absolute numbers and the frequencies of CD4+Foxp3+ Treg in lymphoid organs. Thus, CTX applied in a repetitive high-dose regimen induced a diminution of the endogenous Treg pool size, most likely by inhibiting the homeostatic proliferation of Treg. In the context of the clinical application of a Treg-based immunotherapy, this finding therefore, provides an additional rationale for a therapeutic supplementation with Treg after treatment with CTX in order to compensate for the gap in the numbers of endogenous Treg induced by CTX. On the other hand, these data also indicate that a sufficient time interval between a CTX treatment and the consecutive adoptive Treg transfer must be allowed to avoid interferences between transferred Treg and CTX.

Adoptive transfer of Treg was shown to be effective in influencing the course of murine lupus in a prophylactic and also in a therapeutic setting [21,26]. In previous experiments we found that exclusive transfer of 5 × 10^5 ^CD4+Foxp3+CD25+ Treg was capable of delaying disease progression und prolonging survival in (NZBxNZW) F1 mice with active disease [21]. In these experiments CD4+Foxp3+CD25+ Treg were pre-activated by short-term stimulation with anti-CD3/CD28 in the presence of IL-2 prior to adoptive transfer, because of the IL-2-deprived environment present in mice with active disease, which may impair homeostasis and survival of the transferred Treg in the recipients [21,46]. However, *in vitro *pre-activation of Treg bears the risk that contaminating CD25+ Tcon are also expanded during the *in vitro *stimulation. Thus, in this study, in order to come closer to a clinically applicable setting with the respective need for safety, we omitted the activation step with anti-CD3/CD28 and transferred CD4+Foxp3+CD25+ Treg only after a short incubation time with rIL-2. Transferring the same amount of the now resting Treg, we detected neither a significant clinical response nor a reduction in mortality in addition to the GC/CTX treatment. A significant further reduction in nephritis parameters and mortality was only evident when a 3-fold larger amount of Treg was transferred. Although Treg have been incubated with rIL-2, we suggest that transferred Treg will still be affected by the lack of IL-2 in the recipients, and thus, be impaired in their proper engraftment. This may explain why relatively large amounts of resting Treg are required for eliciting a clinical response. In addition, we cannot rule out that the transferred Treg might also be affected by low amounts of CTX still circulating in the body of the recipients, though we performed Treg transfers five days after the last CTX injection to avoid interferences with CTX. Nevertheless, a significant increase of the Treg frequency compared to the levels before transfer, and higher levels of CD4+Foxp3+ Treg were detectable in the peripheral blood up to four weeks after transfer, implicating that a reasonable number of these cells survived in recipients despite the insufficient availability of IL-2. Concerning the therapeutic durability of the Treg treatment, we suggest that repetition of the Treg transfers, for example, in intervals of two weeks, could sustain remission for a longer period of time. In addition, the efficacy of Treg transfers could probably be increased by simultaneous provision of IL-2 *in vivo *to facilitate their survival and homeostasis in the IL-2-deficient recipients [21].

Another important technical challenge for the clinical implementation of Treg-based therapy in SLE is related to the fact that most SLE patients are lymphopenic and deficient in Treg, which implies that *in vitro *expansion of purified peripheral blood Treg will be unavoidable to obtain sufficient numbers for an autologous Treg transfer. Nevertheless, for safety reasons, we suggest that expanded Treg should be not be transferred before they enter the resting phase after the initial stimulation with anti-CD3/anti-CD28/IL-2.

Apart from the clinical response with a marked reduction in leukocyturia and mortality and the associated increase in the Treg frequency after the additional transfer, analysis of other cellular and humoral parameters in peripheral blood unfortunately did not provide clear suggestions as to how the transferred Treg prolong disease remission. Interestingly, we observed that the CTX-induced decline in PC is more pronounced in the Treg-treated mice suggesting an additive inhibitory effect of the Treg therapy on PC generation. This may serve as supplementary evidence for the recent finding that Treg are able to interfere with the B cell compartment in SLE [47]. Conversely, we observed no additional reduction of autoantibody levels after the Treg transfer, consistent with previous studies by us and other workers on Treg transfers in murine lupus [21,26]. However, as shown here, the analysis of cells from peripheral blood might provide only restricted information and apparently might not always reflect the situation in the lymphoid organs and inflamed tissues where the autoimmune response takes places. With regard to this, more detailed analyses including the lymphoid organs are required, to allow assessment of the mechanisms and the efficacy of transferred Treg at a cellular level.

## Conclusions

In summary, CD4+Foxp3+ Treg were able to prolong disease remission induced by conventional immunosuppressive drugs. Nevertheless, high numbers of transferred Treg were required to elicit a clinical effect. This study supports a role for Treg in the maintenance of SLE remission and provides important rationales and valuable information for the future design of pre-clinical and clinical studies, involving the combination of conventional immunosuppression with a transfer of autologous Treg.

## Abbreviations

APC: allophycocyanin; BrdU: 5-Bromo-2'-deoxyuridine; BSA: bovine serum albumin; CTX: cyclophosphamide; ELISA: enzyme-linked immunosorbent assay; FCS: fetal calf serum; FITC: fluorescein isothiocyanate; GC: glucocorticosteroids; IL: interleukin; PBS: phosphate-buffered saline; PB: plasma blast; PC: plasma cell; PE: phycoerythrin; PerCP: Peridinin-Chlorophyll-Protein Complex; SLE: systemic lupus erythematosus; Tcon: conventional T cells; Treg: regulatory T cells.

## Competing interests

The authors declare that they have no competing interests.

## Authors' contributions

OW carried out the cell sorting and cell culture, performed the mouse studies, acquired data by flow cytometry and participated in data analysis. CS participated in cell sorting and cell culture and acquired data by flow cytometry. RU and LK participated in the mouse studies and acquired data by flow cytometry. JH designed the study, participated in data analysis, performed the statistical analysis and drafted the manuscript. GR participated in the study design and the drafting of the manuscript. All authors read and approved the final manuscript.

## References

[B1] Mok CC Lau CS Pathogenesis of systemic lupus erythematosus J Clin Pathol 2003 56 481 490 10.1136/jcp.56.7.481 12835292 PMC1769989

[B2] Kotzin BL Systemic lupus erythematosus Cell 1996 85 303 306 10.1016/S0092-8674(00)81108-3 8616885

[B3] Tsokos GC Systemic lupus erythematosus N Engl J Med 2011 365 2110 2121 10.1056/NEJMra1100359 22129255

[B4] Riemekasten G Hahn BH Key autoantigens in SLE Rheumatology (Oxford) 2005 44 975 982 10.1093/rheumatology/keh688 15901907

[B5] Crispin JC Liossis SN Kis-Toth K Lieberman LA Kyttaris VC Juang YT Tsokos GC Pathogenesis of human systemic lupus erythematosus: recent advances Trends Mol Med 2010 16 47 57 10.1016/j.molmed.2009.12.005 20138006 PMC2823952

[B6] Albert DA Hadler NM Ropes MW Does corticosteroid-therapy affect the survival of patients with systemic lupus-erythematosus Arthritis Rheum 1979 22 945 953 10.1002/art.1780220901 475873

[B7] Lo MS Tsokos GC Treatment of systemic lupus erythematosus: new advances in targeted therapy Ann N Y Acad Sci 2012 1247 138 152 10.1111/j.1749-6632.2011.06263.x 22236448

[B8] Austin HA Klippel JH Balow JE le Riche NG Steinberg AD Plotz PH Decker JL Therapy of lupus nephritis. Controlled trial of prednisone and cytotoxic drugs N Engl J Med 1986 314 614 619 10.1056/NEJM198603063141004 3511372

[B9] Steinberg AD Kaltreider HB Staples PJ Goetzl EJ Talal N Decker JL Cyclophosphamide in lupus nephritis: a controlled trial Ann Intern Med 1971 75 165 171 4104337 10.7326/0003-4819-75-2-165

[B10] Steinberg AD Steinberg SC Long-term preservation of renal function in patients with lupus nephritis receiving treatment that includes cyclophosphamide versus those treated with prednisone only Arthritis Rheum 1991 34 945 950 10.1002/art.1780340803 1859488

[B11] Karim Y D'Cruz DP The NIH pulse cyclophosphamide regime: the end of an era? Lupus 2004 13 1 3 10.1191/0961203304lu524ed 14870910

[B12] Fontenot JD Gavin MA Rudensky AY Foxp3 programs the development and function of CD4+CD25+ regulatory T cells Nat Immunol 2003 4 330 336 12612578 10.1038/ni904

[B13] Sakaguchi S Naturally arising Foxp3-expressing CD25+CD4+ regulatory T cells in immunological tolerance to self and non-self Nat Immunol 2005 6 345 352 15785760 10.1038/ni1178

[B14] Kim JM Rasmussen JP Rudensky AY Regulatory T cells prevent catastrophic aautoimmunity throughout the lifespan of mice Nat Immunol 2007 8 191 197 10.1038/ni1428 17136045

[B15] Bluestone JA Regulatory T-cell therapy: is it ready for the clinic? Nat Rev Immunol 2005 5 343 349 10.1038/nri1574 15775994

[B16] Humrich J Riemekasten G [Regulatory T cells in rheumatic diseases] Dtsch Med Wochenschr 2006 131 2288 2291 10.1055/s-2006-951367 17036273

[B17] Taams LS Palmer DB Akbar AN Robinson DS Brown Z Hawrylowicz CM Regulatory T cells in human disease and their potential for therapeutic manipulation Immunology 2006 118 1 9 10.1111/j.1365-2567.2006.02348.x 16630018 PMC1782265

[B18] Brusko TM Putnam AL Bluestone JA Human regulatory T cells: role in autoimmune disease and therapeutic opportunities Immunol Rev 2008 223 371 390 10.1111/j.1600-065X.2008.00637.x 18613848

[B19] Costantino CM Baecher-Allan CM Hafler DA Human regulatory T cells and autoimmunity Eur J Immunol 2008 38 921 924 10.1002/eji.200738104 18395861 PMC2752283

[B20] Wing K Sakaguchi S Regulatory T cells exert checks and balances on self tolerance and autoimmunity Nat Immunol 2010 11 7 13 20016504 10.1038/ni.1818

[B21] Humrich JY Morbach H Undeutsch R Enghard P Rosenberger S Weigert O Kloke L Heimann J Gaber T Brandenburg S Scheffold A Huehn J Radbruch A Burmester GR Riemekasten G Homeostatic imbalance of regulatory and effector T cells due to IL-2 deprivation amplifies murine lupus Proc Natl Acad Sci USA 2010 107 204 209 10.1073/pnas.0903158107 20018660 PMC2806746

[B22] Valencia X Yarboro C Illei G Lipsky PE Deficient CD4+CD25high T regulatory cell function in patients with active systemic lupus erythematosus J Immunol 2007 178 2579 2588 17277168 10.4049/jimmunol.178.4.2579

[B23] Liu MF Wang CR Fung LL Wu CR Decreased CD4+CD25+ T cells in peripheral blood of patients with systemic lupus erythematosus Scand J Immunol 2004 59 198 202 10.1111/j.0300-9475.2004.01370.x 14871297

[B24] Zhang B Zhang X Tang FL Zhu LP Liu Y Lipsky PE Clinical significance of increased CD4+CD25-Foxp3+ T cells in patients with new-onset systemic lupus erythematosus Ann Rheum Dis 2008 67 1037 1040 18199598 10.1136/ard.2007.083543

[B25] Miyara M Amoura Z Parizot C Badoual C Dorgham K Trad S Nochy D Debre P Piette JC Gorochov G Global natural regulatory T cell depletion in active systemic lupus erythematosus J Immunol 2005 175 8392 8400 16339581 10.4049/jimmunol.175.12.8392

[B26] Scalapino KJ Tang Q Bluestone JA Bonyhadi ML Daikh DI Suppression of disease in New Zealand Black/New Zealand White lupus-prone mice by adoptive transfer of ex vivo expanded regulatory T cells J Immunol 2006 177 1451 1459 16849451 10.4049/jimmunol.177.3.1451

[B27] Bonelli M Savitskaya A Steiner CW Rath E Smolen JS Scheinecker C Phenotypic and functional analysis of CD4+ CD25- Foxp3+ T cells in patients with systemic lupus erythematosus J Immunol 2009 182 1689 1695 19155519 10.4049/jimmunol.182.3.1689

[B28] Alexander T Thiel A Rosen O Massenkeil G Sattler A Kohler S Mei H Radtke H Gromnica-Ihle E Burmester GR Arnold R Radbruch A Hiepe F Depletion of autoreactive immunologic memory followed by autologous hematopoietic stem cell transplantation in patients with refractory SLE induces long-term remission through de novo generation of a juvenile and tolerant immune system Blood 2009 113 214 223 10.1182/blood-2008-07-168286 18824594

[B29] Shlomchik MJ Craft JE Mamula MJ From T to B and back again: positive feedback in systemic autoimmune disease Nat Rev Immunol 2001 1 147 153 10.1038/35100573 11905822

[B30] Hofer T Muehlinghaus G Moser K Yoshida T H EM Hebel K Hauser A Hoyer B E OL Dorner T Manz RA Hiepe F Radbruch A Adaptation of humoral memory Immunol Rev 2006 211 295 302 10.1111/j.0105-2896.2006.00380.x 16824136

[B31] Crispin JC Kyttaris VC Terhorst C Tsokos GC T cells as therapeutic targets in SLE Nat Rev Rheumatol 2010 6 317 325 10.1038/nrrheum.2010.60 20458333 PMC2924434

[B32] Hiepe F Dorner T Hauser AE Hoyer BF Mei H Radbruch A Long-lived autoreactive plasma cells drive persistent autoimmune inflammation Nat Rev Rheumatol 2011 7 170 178 21283146 10.1038/nrrheum.2011.1

[B33] Hoyer BF Moser K Hauser AE Peddinghaus A Voigt C Eilat D Radbruch A Hiepe F Manz RA Short-lived plasmablasts and long-lived plasma cells contribute to chronic humoral autoimmunity in NZB/W mice J Exp Med 2004 199 1577 1584 10.1084/jem.20040168 15173206 PMC2211779

[B34] Schiffer L Sinha J Wang X Huang W von Gersdorff G Schiffer M Madaio MP Davidson A Short term administration of costimulatory blockade and cyclophosphamide induces remission of systemic lupus erythematosus nephritis in NZB/W F1 mice by a mechanism downstream of renal immune complex deposition J Immunol 2003 171 489 497 12817034 10.4049/jimmunol.171.1.489

[B35] Theofilopoulos AN Dixon FJ Murine models of systemic lupus erythematosus Adv Immunol 1985 37 269 390 3890479 10.1016/s0065-2776(08)60342-9

[B36] Riemekasten G Langnickel D Enghard P Undeutsch R Humrich J Ebling FM Hocher B Humaljoki T Neumayer H Burmester GR Hahn BH Radbruch A Hiepe F Intravenous injection of a D1 protein of the Smith proteins postpones murine lupus and induces type 1 regulatory T cells J Immunol 2004 173 5835 5842 15494537 10.4049/jimmunol.173.9.5835

[B37] Bagley CM Jr Bostick FW DeVita VT Jr Clinical pharmacology of cyclophosphamide Cancer Res 1973 33 226 233 4688880

[B38] Joy MS La M Wang J Bridges AS Hu Y Hogan SL Frye RF Blaisdell J Goldstein JA Dooley MA Brouwer KL Falk RJ Cyclophosphamide and 4-hydroxycyclophosphamide pharmacokinetics in patients with glomerulonephritis secondary to lupus and small vessel vasculitis Br J Clin Pharmacol 2012 74 445 455 10.1111/j.1365-2125.2012.04223.x 22380717 PMC3477346

[B39] Cao Y Zhao J Yang Z Cai Z Zhang B Zhou Y Shen GX Chen X Li S Huang B CD4+FOXP3+ regulatory T cell depletion by low-dose cyclophosphamide prevents recurrence in patients with large condylomata acuminata after laser therapy Clin Immunol 2010 136 21 29 10.1016/j.clim.2010.02.020 20338811

[B40] Greten TF Ormandy LA Fikuart A Hochst B Henschen S Horning M Manns MP Korangy F Low-dose cyclophosphamide treatment impairs regulatory T cells and unmasks AFP-specific CD4+ T-cell responses in patients with advanced HCC J Immunother 2010 33 211 218 10.1097/CJI.0b013e3181bb499f 20139774

[B41] Lutsiak ME Semnani RT De Pascalis R Kashmiri SV Schlom J Sabzevari H Inhibition of CD4(+)25+ T regulatory cell function implicated in enhanced immune response by low-dose cyclophosphamide Blood 2005 105 2862 2868 10.1182/blood-2004-06-2410 15591121

[B42] Takeuchi A Eto M Yamada H Tatsugami K Naito S Yoshikai Y A reduction of recipient regulatory T cells by cyclophosphamide contributes to an anti-tumor effect of nonmyeloablative allogeneic stem cell transplantation in mice Int J Cancer 2011 130 365 376 21351096 10.1002/ijc.26009

[B43] Zhao J Cao Y Lei Z Yang Z Zhang B Huang B Selective depletion of CD4+CD25+Foxp3+ regulatory T cells by low-dose cyclophosphamide is explained by reduced intracellular ATP levels Cancer Res 2010 70 4850 4858 10.1158/0008-5472.CAN-10-0283 20501849

[B44] Brode S Cooke A Immune-potentiating effects of the chemotherapeutic drug cyclophosphamide Crit Rev Immunol 2008 28 109 126 10.1615/CritRevImmunol.v28.i2.20 18540827

[B45] Enghard P Humrich JY Rudolph B Rosenberger S Biesen R Kuhn A Manz R Hiepe F Radbruch A Burmester GR Riemekasten G CXCR3+CD4+ T cells are enriched in inflamed kidneys and urine and provide a new biomarker for acute nephritis flares in systemic lupus erythematosus patients Arthritis Rheum 2009 60 199 206 10.1002/art.24136 19116922

[B46] Fontenot JD Rasmussen JP Gavin MA Rudensky AY A function for interleukin 2 in Foxp3-expressing regulatory T cells Nat Immunol 2005 6 1142 1151 10.1038/ni1263 16227984

[B47] Iikuni N Lourenco EV Hahn BH La Cava A Cutting edge: Regulatory T cells directly suppress B cells in systemic lupus erythematosus J Immunol 2009 183 1518 22 10.4049/jimmunol.0901163 19570829 PMC2730469

